# Anterior Segment Measurements in Normal Cats Using Ultrasound Biomicroscopy

**DOI:** 10.3390/vetsci13010050

**Published:** 2026-01-06

**Authors:** Donghee Kim, Myeongjee Kwon, Ji Seung Jung, Jiyi Hwang, Sooyeon Lee, Mirae Lee, Haemi Seol, Kyung-Mee Park

**Affiliations:** Laboratory of Veterinary Surgery and Ophthalmology, College of Veterinary Medicine, Chungbuk National University, Cheongju 28644, Republic of Korea; kdh087@gmail.com (D.K.); k3clover@naver.com (M.K.); wjdwltmd00@gmail.com (J.S.J.); wldml1013@gmail.com (J.H.); waterkite0921@gmail.com (S.L.); dyj06096@gmail.com (M.L.); paratu091@gmail.com (H.S.)

**Keywords:** ultrasound biomicroscopy, feline anterior segment, anterior chamber, ciliary body, ciliary cleft

## Abstract

Establishing baseline ultrasound biomicroscopy (UBM) measurements of the feline anterior segment is essential for accurate evaluation of ocular diseases. This study aimed to establish normative UBM parameters in clinically normal cats and to assess the influence of sex, body weight, and age on these measurements. Twenty eyes from twenty healthy adult cats were examined using a standardized UBM protocol under general anesthesia to ensure consistent positioning and optimal visualization. Quantitative measurements were obtained for corneal, anterior chamber, iris, ciliary cleft, and ciliary body parameters. The mean perilimbal corneal thickness was 0.59 mm, anterior chamber depth was 4.11 mm, and peripheral anterior chamber depth was 3.11 mm. The angle-opening distance and iridocorneal angle measured 1.73 mm and 28.89°, respectively. Ciliary cleft parameters included a width of 1.22 mm, length of 2.07 mm, and area of 1.10 mm^2^. Longitudinal and combined longitudinal–radial ciliary body thicknesses were 0.52 mm and 0.78 mm, respectively, while ciliary body axial length and ciliary process scleral angle averaged 2.01 mm and 58.98°. Iris base width and iris middle width measured 0.35 mm and 0.54 mm, respectively. Sex-based analysis revealed significant differences in peripheral anterior chamber depth and ciliary cleft width, whereas body weight showed positive correlations with iris thickness parameters. No significant associations were identified between age and any anterior segment measurements. These findings establish comprehensive normative UBM reference values for the feline anterior segment and provide a quantitative anatomical framework for future clinical and disease-oriented studies in feline ophthalmology.

## Simple Summary

Cats can develop a variety of ocular diseases, but early diagnosis is often challenging because detailed reference information on the normal internal structure of the feline eye is limited. This study used ultrasound biomicroscopy, a high-resolution imaging technique, to quantitatively evaluate the anterior segment of healthy cats, including the cornea, anterior chamber, iris, and ciliary body. In addition to establishing normal reference values, we assessed whether these measurements were influenced by sex, body weight, or age. Most parameters were consistent across these factors, although limited associations were identified for specific iris-related and anterior chamber measurements. These normative data provide a practical reference for veterinarians and may support earlier detection and more accurate evaluation of anterior segment disorders such as glaucoma, uveitis, and iris-related diseases in cats.

## 1. Introduction

The anterior segment of the feline eye has several distinct anatomical features. One notable characteristic is the considerable depth of the anterior chamber, which differs from that of dogs [1,2]. This increased depth is primarily due to the more posterior positioning of the lens [3]. Additionally, the geometric iridocorneal angle (ICA), where the iris meets the cornea, is significantly wider in cats [1,4]. Thus, cats display anatomical features that differ from those of both dogs and humans. Several studies have applied ultrasound biomicroscopy to evaluate the feline anterior segment, primarily in the context of pharmacologic interventions or comparative analyses across species. In these investigations, baseline anterior segment measurements were reported to support specific experimental aims rather than to establish comprehensive normative reference values.

Ultrasound biomicroscopy(UBM) is an advanced imaging technique utilizing high frequency transducers (35–100 MHz), with axial accuracy up to 20 μm and lateral resolution up to 50 μm [5,6]. Although its tissue penetration is limited to 4–5 mm, UBM employs a higher frequency transducer that delivers enhanced resolution of the anterior segment compared to traditional B-scan ultrasonography [5,7]. This allows for precise visualization of the cornea, iris, ciliary body, and lens, structures often difficult to evaluate with lower-frequency techniques [8]. UBM is particularly useful in diagnosing feline anterior segment diseases, such as secondary glaucoma due to uveitis by measuring the ICA [9,10]. Additionally, it can aid in detecting iris thickening associated with diffuse iris melanoma and lymphoma, conditions that are difficult to diagnose using conventional B-scan or slit-lamp biomicroscopy [11].

Several studies have applied ultrasound biomicroscopy to evaluate the feline anterior segment, primarily in the context of pharmacologic interventions or comparative analyses across species. In particular, our research group has previously reported UBM-derived anterior segment measurements in clinically normal cats as control data in multiple studies [12,13,14]. In those investigations, baseline measurements were presented to support specific experimental objectives rather than to establish comprehensive normative reference standards.

Therefore, the objective of the present study was to establish a comprehensive and systematically integrated normative reference framework for the feline anterior segment, encompassing corneal, anterior chamber, iris, ciliary cleft, and ciliary body parameters derived from a single, well-defined cohort of clinically normal cats under standardized imaging conditions, and to evaluate the potential influence of sex, body weight, and age on these measurements.

## 2. Materials and Methods

### 2.1. Clinical Information

This prospective study was conducted at the Veterinary Medical Teaching Hospital of Chungbuk National University, Cheongju, South Korea, between November 2022 and August 2024. A total of 20 Domestic Shorthair cats (20 eyes) were enrolled after comprehensive ophthalmological examinations were performed by qualified personnel under the supervision of veterinary ophthalmology faculty to ensure normal ocular conditions. The sample comprised 9 neutered males and 11 spayed females, with a mean weight of 5.32 ± 0.85 kg (range: 3.8–7.2 kg) and a mean age of 5.5 ± 3.15 years (range: 2–13 years). Intraocular pressure (IOP) was measured in all eyes, yielding a mean value of 18.65 ± 2.02 mmHg (range: 13–21 mmHg). Of the eyes evaluated, 12 were left eyes (OS) and 8 were right eyes (OD). Data collection was performed on patients with normal ocular conditions, and informed consent was obtained from the owners of all cats. Ethical approval for the study was provided by the Institutional Animal Care and Use Committee (CBNUA-2040-22-02).

### 2.2. Ophthalmological Evaluations

Comprehensive ophthalmic evaluations were performed using various diagnostic techniques to assess ocular health. These evaluations were conducted by veterinarians specializing in ophthalmology who are enrolled in doctoral programs, ensuring that all examinations met the criteria for normal ocular health. These included slit-lamp biomicroscopy (MW50D, SHIGIYA, Hiroshima, Japan) and the Schirmer Tear Test (Schirmer Tear Flow Strips, Gulden Ophthalmics, Elkins Park, PA, USA) to measure tear production. Reflex assessments, including the menace response, pupillary light reflex, and dazzle reflex, were also conducted. Intraocular pressure (IOP) was measured with a rebound tonometer (TonoVet Plus^®^, icare, Vantaa, Finland). Gonioscopy was performed using Ocular Koeppe Diagnostic Lenses (Ocular Instruments Inc., Bellevue, WA, USA). Fundus examinations were excluded from this study, as pupil dilation was not permitted in the protocol. Only eyes deemed normal based on these tests were included in the study.

### 2.3. UBM Examination

UBM was performed with the VuPAD^®^ (Sonomed Escalon, Lake Success, NY, USA) using a transparent thin film probe cover filled with distilled water (ClearScan^®^, ESL, Inc., Plymouth, MN, USA), providing detailed visualization of the anterior segment structures. The patients were given 100 mg of gabapentin an hour before the procedure to reduce anxiety. Unlike in dogs, UBM measurements in cats were performed under anesthesia because, due to their relatively larger corneas and reduced scleral exposure, complete ocular immobilization is crucial for adequately visualizing the limbus. Anesthesia was induced with 6 mg/kg propofol (Freepol-MCT inj., Daewon Pharm. Co., Ltd., Sungdong-gu, Republic of Korea) intravenously and maintained with isoflurane (Terrell^®^, Piramal Critical Care Inc., Bethlehem, PA, USA). During the UBM procedure, all cats were kept in a dorsal position. The lighting conditions in the operating room were dim and consistent for all animals throughout the study. Topical proparacaine (Paracaine Eye Drops 0.5%) was applied to the ocular surface.

After induction of general anesthesia, cats were positioned in dorsal recumbency. The upper eyelid was gently elevated, and a Barraquer eyelid speculum was applied to ensure consistent exposure of the scleral surface. The UBM probe was positioned perpendicular to the limbus, and scans were obtained at the superotemporal quadrant corresponding to the 12-o’clock limbal position using angle-detail mode.

For each eye, a minimum of three UBM images were acquired. Images that clearly visualized the iridocorneal angle, ciliary cleft, and ciliary body without motion artifacts were selected for analysis. Quantitative measurements were obtained from each selected image, and the mean value of at least three measurements was calculated for each parameter. All measurements were performed by a single experienced examiner using standardized anatomical landmarks to minimize measurement variability.

### 2.4. Measurement Parameters

**Figure 1:**
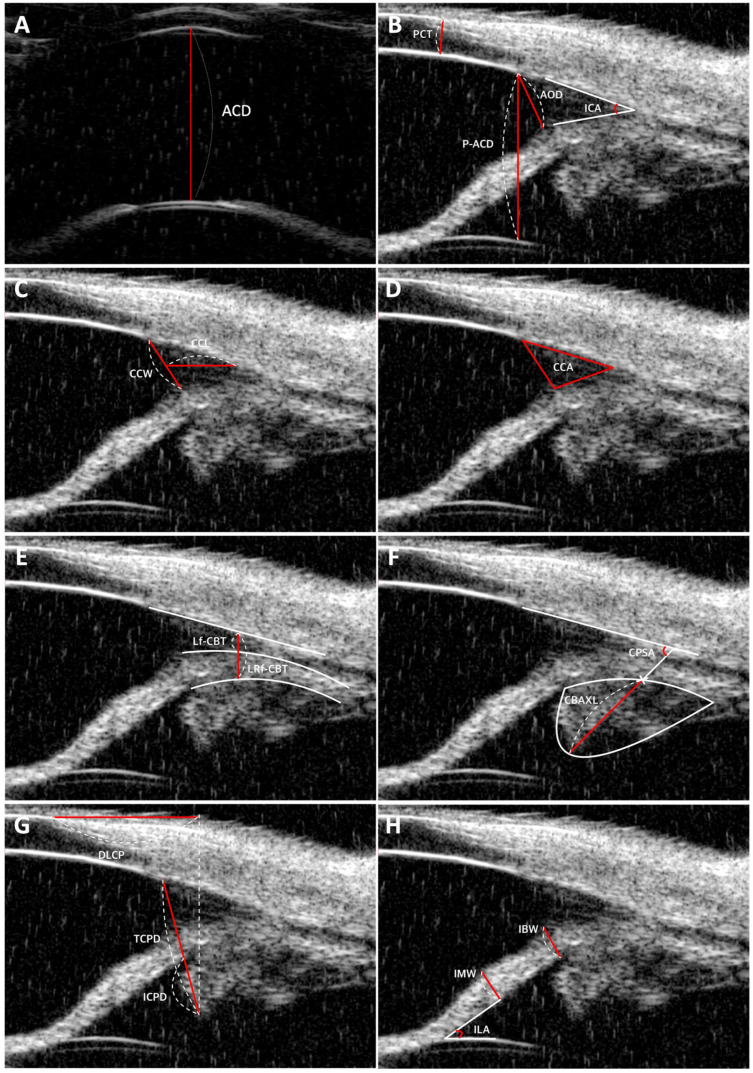
Representative ultrasound biomicroscopy (UBM) images of anterior segment parameters in normal feline eyes. (**A**) Anterior chamber depth (ACD). (**B**) perilimbal corneal thickness (PCT), peripheral anterior chamber depth (P-ACD), angle-opening distance (AOD), iridocorneal angle (ICA). (**C**) Ciliary cleft width (CCW), and ciliary cleft length (CCL). (**D**) Ciliary cleft area (CCA). (**E**) Longitudinal fibers of ciliary body thickness (Lf-CBT) and longitudinal and radial fibers of ciliary body thickness (LRf-CBT). (**F**) Ciliary body axial length (CBAXL) and ciliary process scleral angle (CPSA). (**G**) Distance from limbus to the first ciliary process (DLCP), trabecular–ciliary process distance (TCPD), and iris–ciliary process distance (ICPD). (**H**) Iris base width (IBW), iris middle width (IMW), and iris–lens angle (ILA). Red lines indicate the measured parameters; white dashed lines represent the length or angle corresponding to the red lines; and white solid lines indicate reference guidelines.

This study employed UBM to quantitatively assess a range of parameters within the feline anterior segment. A comprehensive list of these parameters is provided in Table 1, along with detailed descriptions of the measurement procedures. The measured parameters included perilimbal corneal thickness (PCT), anterior chamber depth (ACD), peripheral anterior chamber depth (P-ACD), angle-opening distance (AOD), iridocorneal angle (ICA), ciliary cleft width (CCW), ciliary cleft length (CCL), ciliary cleft area (CCA), longitudinal region of ciliary body thickness (Lf-CBT), combined longitudinal and radial region of ciliary body thickness (LRf-CBT), ciliary body axial length (CBAXL), ciliary process scleral angle (CPSA), distance from limbus to first ciliary process (DLCP), trabecular–ciliary process distance (TCPD), iris–ciliary process distance (ICPD), iris base width (IBW), iris middle width (IMW), and iris–lens angle (ILA). Corresponding figures illustrating the methodology are also included in Table 1 and Figure 1.

### 2.5. Statistical Analysis

Statistical analyses were performed using GraphPad Prism (GraphPad Software Version 10.6.1, San Diego, CA, USA). Descriptive statistics, including the mean, standard deviation, range, and 95% confidence intervals, were calculated for all UBM-derived parameters and are presented in Table 1. Sex-based differences were evaluated using two-tailed Mann–Whitney U tests. Associations between body weight or age and each UBM parameter were assessed using Spearman’s rank correlation analysis. Normality of data distribution was assessed using the Shapiro–Wilk test. All statistical analyses and graphical representations were generated using GraphPad Prism. A *p*-value < 0.05 was considered statistically significant.

## 3. Results

### 3.1. Baseline Clinical and Neuro-Ophthalmic Status of the Study Population

All cats included in the analysis were confirmed to be clinically and neuro-ophthalmologically normal based on comprehensive pre-examination screening, including normal menace response, pupillary light reflex, and dazzle reflex. The results presented below therefore reflect baseline UBM measurements obtained from eyes without detectable functional abnormalities.

### 3.2. Anterior Segment Measurements

All anterior segment measurements obtained using ultrasound biomicroscopy—including mean values, standard deviations, and ranges—are summarized in Table 2.

### 3.3. Sex-Based Differences in Anterior Segment Parameters

**Figure 2:**
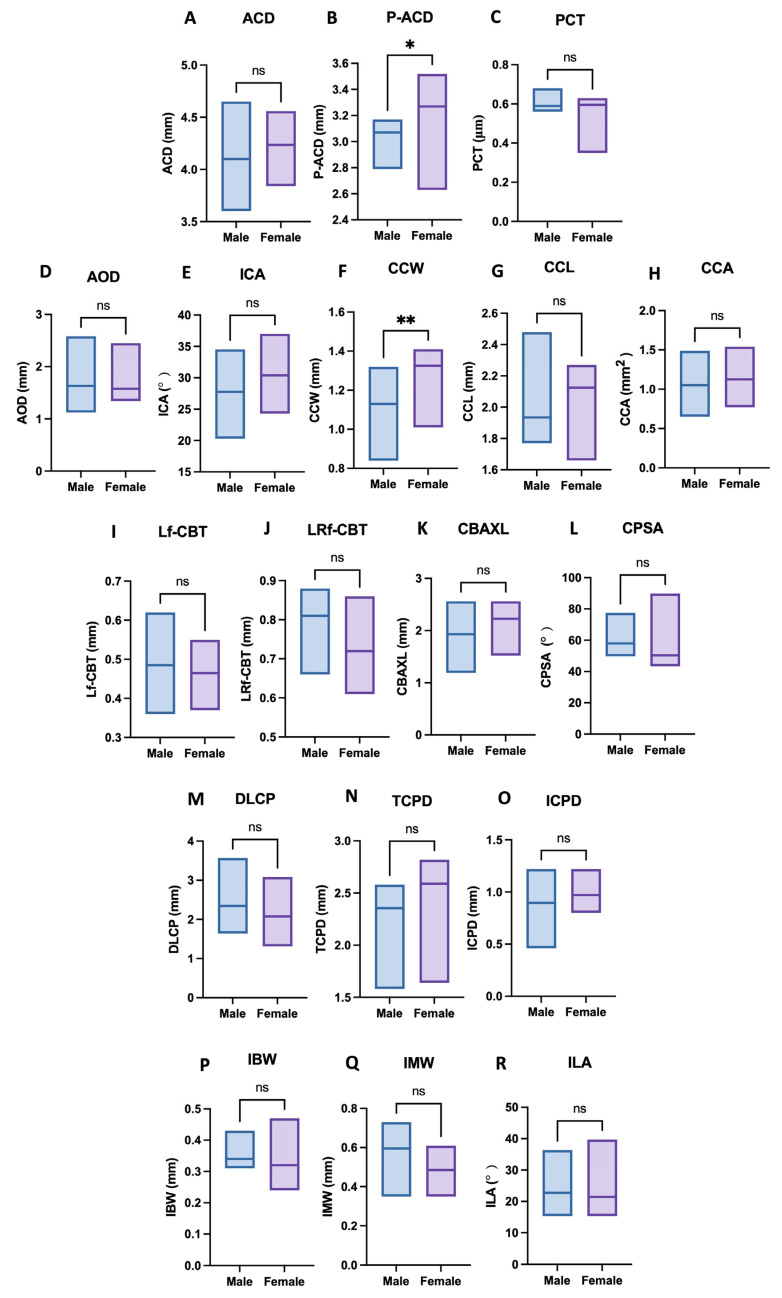
Sex-based comparison of ultrasound biomicroscopy parameters in normal cats. Box-and-whisker plots illustrate sex-based distributions of anterior segment parameters measured by ultrasound biomicroscopy (UBM) in normal cats. Parameters include anterior chamber depth (ACD, (**A**)), peripheral anterior chamber depth (P-ACD, (**B**)), perilimbal corneal thickness (PCT, (**C**)), angle-opening distance (AOD, (**D**)), iridocorneal angle (ICA, (**E**)), ciliary cleft width (CCW, (**F**), ciliary cleft length (CCL, (**G**)), ciliary cleft area (CCA, (**H**)), longitudinal region of ciliary body thickness (Lf-CBT, (**I**)), combined longitudinal and radial region of ciliary body thickness (LRf-CBT, (**J**)), ciliary body axial length (CBAXL, (**K**)), ciliary process scleral angle (CPSA, (**L**)), distance from limbus to the first ciliary process (DLCP, (**M**)), trabecular–ciliary process distance (TCPD, (**N**)), iris–ciliary process distance (ICPD, (**O**)), iris base width (IBW, (**P**)), iris middle width (IMW, (**Q**)), and iris–lens angle (ILA, (**R**)). Boxes represent the interquartile range with median values indicated, and whiskers denote the minimum and maximum values. Statistically significant sex-related differences were observed in P-ACD and CCW (two-tailed Mann–Whitney U tests), whereas all other parameters showed no significant differences between male and female cats. * *p* < 0.05; ** *p* < 0.01; ns, not significant.

Sex-based comparisons revealed statistically significant differences in peripheral anterior chamber depth (P-ACD) and ciliary cleft width (CCW). Female cats exhibited greater P-ACD values than male cats (*p* = 0.0488), and CCW was also significantly larger in female cats (*p* = 0.0085). In contrast, no significant sex-related differences were observed in any of the remaining corneal, anterior chamber, iris, or ciliary body parameters (*p* > 0.05). Sex-based distributions of these parameters are illustrated in Figure 2.

### 3.4. Associations Between Body Weight and Anterior Segment Parameters

**Figure 3:**
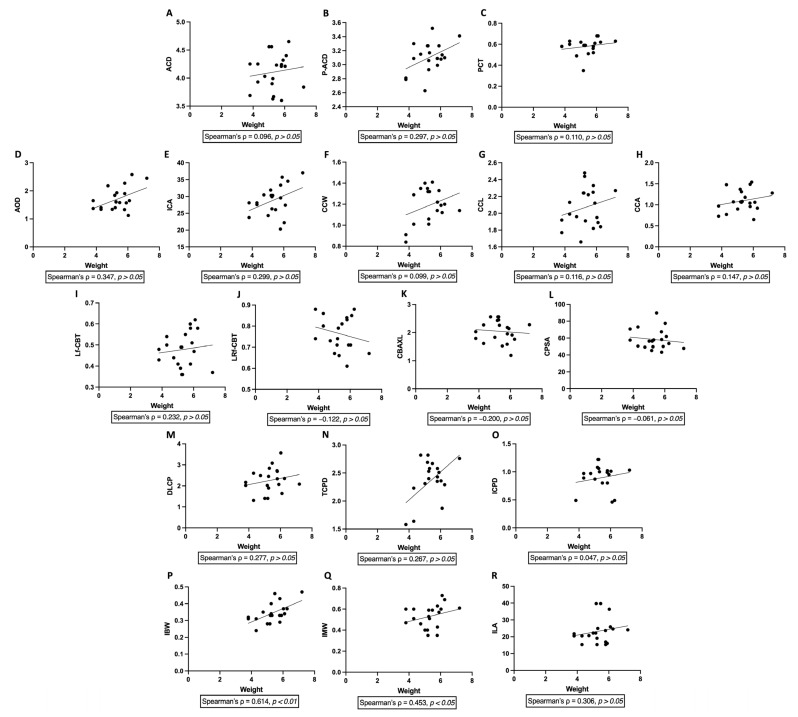
Correlations between body weight and ultrasound biomicroscopy parameters in normal cats. Scatter plots illustrate the relationships between body weight and anterior segment parameters measured by ultrasound biomicroscopy (UBM) in normal cats. Parameters include anterior chamber depth (ACD, (**A**)), peripheral anterior chamber depth (P-ACD, (**B**)), perilimbal corneal thickness (PCT, (**C**)), angle-opening distance (AOD, (**D**)), iridocorneal angle (ICA, (**E**)), ciliary cleft width (CCW, (**F**)), ciliary cleft length (CCL, (**G**)), ciliary cleft area (CCA, (**H**)), longitudinal region of ciliary body thickness (Lf-CBT, (**I**)), combined longitudinal and radial region of ciliary body thickness (LRf-CBT, (**J**)), ciliary body axial length (CBAXL, (**K**)), ciliary process scleral angle (CPSA, (**L**)), distance from limbus to the first ciliary process (DLCP, (**M**)), trabecular–ciliary process distance (TCPD, (**N**)), iris–ciliary process distance (ICPD, (**O**)), iris base width (IBW, (**P**)), iris middle width (IMW, (**Q**)), and iris–lens angle (ILA, (**R**)). Spearman correlation analysis demonstrated significant positive correlations between body weight and iris thickness parameters, including IBW (ρ = 0.614, *p* = 0.0040) and IMW (ρ = 0.453, *p* = 0.0448), whereas no significant correlations were observed between body weight and the remaining anterior segment parameters (*p* > 0.05). Lines represent fitted trends for visualization purposes only.

Spearman correlation analysis revealed significant positive correlations between body weight and iris thickness parameters, including iris base width (IBW; Spearman’s ρ = 0.614, *p* = 0.0040) and iris middle width (IMW; ρ = 0.453, *p* = 0.0448). In contrast, body weight was not significantly correlated with any other corneal, anterior chamber, ciliary cleft, or ciliary body parameters (all *p* > 0.05) (Figure 3).

### 3.5. Associations Between Age and Anterior Segment Parameters

**Figure 4:**
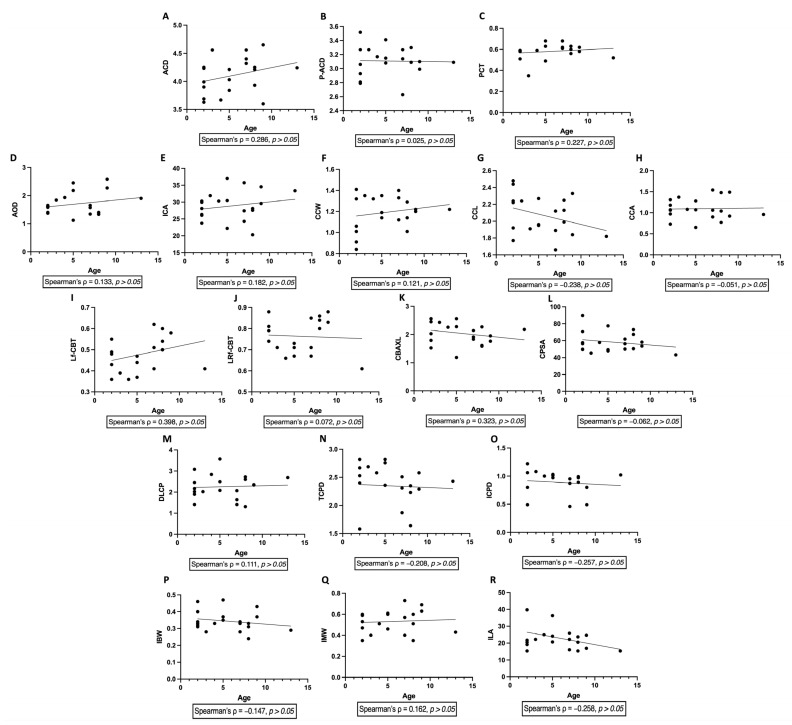
Correlations between age and ultrasound biomicroscopy parameters in normal cats. Scatter plots illustrate the relationships between age and anterior segment parameters measured by ultrasound biomicroscopy (UBM) in normal cats. Parameters include anterior chamber depth (ACD, (**A**)), peripheral anterior chamber depth (P-ACD, (**B**)), perilimbal corneal thickness (PCT, (**C**)), angle-opening distance (AOD, (**D**)), iridocorneal angle (ICA, (**E**)), ciliary cleft width (CCW, (**F**)), ciliary cleft length (CCL, (**G**)), ciliary cleft area (CCA, (**H**)), longitudinal region of ciliary body thickness (Lf-CBT, (**I**)), combined longitudinal and radial region of ciliary body thickness (LRf-CBT, (**J**)), ciliary body axial length (CBAXL, (**K**)), ciliary process scleral angle (CPSA, (**L**)), distance from limbus to the first ciliary process (DLCP, (**M**)), trabecular–ciliary process distance (TCPD, (**N**)), iris–ciliary process distance (ICPD, (**O**)), iris base width (IBW, (**P**)), iris middle width (IMW, (**Q**)), and iris–lens angle (ILA, (**R**)). Spearman correlation analysis demonstrated no significant correlations between age and any of the evaluated parameters (all *p* > 0.05). Lines represent fitted trends for visualization purposes only.

Spearman correlation analysis revealed no significant associations between age and any ultrasound biomicroscopy-derived anterior segment parameters. Specifically, age was not significantly correlated with corneal (PCT), anterior chamber (ACD, P-ACD, AOD, ICA), ciliary cleft (CCW, CCL, CCA), ciliary body (CBAXL, CPSA, DLCP, TCPD), or iris-related parameters (IBW, IMW, ILA) (all *p* > 0.05) (Figure 4). 

## 4. Discussion

This study provides a comprehensive ultrasound biomicroscopy-based characterization of anterior segment structures in clinically normal cats, encompassing corneal, anterior chamber, ciliary cleft, ciliary body, and iris-related parameters. Collectively, these measurements reflect key anatomical components involved in aqueous humor dynamics, anterior chamber configuration, and iris morphology, which are relevant to the pathophysiology of common feline ocular diseases, including secondary glaucoma, chronic uveitis, and iris-related disorders. By establishing standardized reference values across multiple structural domains, the present findings offer an anatomical framework for interpreting subtle anterior segment alterations in future clinical and disease-oriented investigations.

In the present study, UBM examinations were not performed in conjunction with other surgical procedures, such as spay or castration. Dorsal recumbency under general anesthesia was intentionally selected to optimize imaging reproducibility and accessibility of anterior segment structures. In feline eyes, dorsal positioning facilitates stable visualization not only of the superior (12-o’clock) limbal region but also of other anterior segment regions, whereas sternal recumbency primarily provides optimal access to the superior limbus alone. Because the primary objective of this study was to establish normative reference values under highly standardized conditions, body positioning was carefully considered to minimize measurement variability across examinations. Based on our experience, dorsal recumbency provided the most consistent imaging conditions for feline UBM acquisition. Nevertheless, to maintain analytical consistency, only measurements obtained from the 12-o’clock limbal position were included in the final analysis.

At present, direct evidence from human medicine demonstrating posture-dependent differences in ciliary body-specific UBM parameters is scarce, and no studies have systematically compared ciliary body thickness or morphology between sitting and supine positions in humans. However, several human UBM studies have shown that body position significantly influences anterior segment geometry, including angle configuration and lens position. In particular, UBM comparisons between sitting and supine postures have demonstrated narrower anterior chamber angles and increased anterior displacement of the lens in the supine position, indicating that gravitational or posture-related biomechanical factors can alter the spatial relationships of anterior segment structures. These posture-dependent changes in angle and lens configuration suggest that closely related structures, such as the ciliary body and zonular–lens complex, may also be indirectly affected, even if this has not yet been directly quantified in human studies [15,16].

Accordingly, it is reasonable to consider that differences in body position between studies may contribute to discrepancies in reported ciliary body parameter values, particularly when comparing results obtained under different imaging protocols. Nonetheless, because direct human evidence remains limited, this interpretation should be made cautiously. The potential influence of body position on ciliary body measurements represents an important area for future investigation and should be explicitly considered when comparing UBM-derived parameters across studies. In this context, the use of dorsal recumbency in the present study, although uncommon in routine veterinary UBM imaging, should be regarded as a methodological choice aimed at maximizing standardization rather than replicating typical clinical positioning, and this should be acknowledged as a limitation when extrapolating our findings to other imaging conditions.

In the present study, PCT was measured instead of central corneal thickness. Central corneal thickness was not assessed because the use of a UBM scan cover occasionally limited precise delineation of the corneal epithelial boundary in some images. Accordingly, PCT was included as a descriptive parameter to characterize corneal morphology under the applied imaging conditions. ACD and p-ACD were also documented to provide contextual information regarding anterior chamber configuration in clinically normal cats. These measurements are presented descriptively and should be interpreted within the methodological context of the present study, as differences in imaging techniques, tissue handling, and measurement definitions may influence absolute values across studies.

Angle-related parameters, including AOD and ICA, are clinically relevant because secondary glaucoma is relatively common in cats, often associated with chronic uveitis and peripheral anterior synechiae formation [2,17]. In our previous comparative UBM study, normal cats demonstrated relatively large AOD and ICA values compared with dogs, consistent with the lower prevalence of primary angle-closure glaucoma in this species. In the present study, the mean AOD (1.73 mm) and ICA (28.89°) were comparable to those previously reported in clinically normal cats, supporting the reproducibility of these angle-related measurements under standardized conditions [12]. Although the present study was conducted under standardized lighting conditions, these parameters are known to be influenced by iris configuration and pupil size [18]. Therefore, the reported values should be interpreted with caution and are best considered as reference ranges rather than definitive diagnostic thresholds.

Ciliary cleft parameters, including CCW, CCL, and CCA, reflect the anatomical configuration of the aqueous humor outflow pathway. In our previous feline UBM studies, CCW, CCL, and CCA values in normal cats were reported to range approximately from 1.07–1.23 mm, 2.03–2.27 mm, and 1.08–1.15 mm^2^, respectively [12]. The values observed in the present study (CCW: 1.22 mm, CCL: 2.07 mm, CCA: 1.10 mm^2^) are largely consistent with these previously published data, despite being obtained under a different experimental context and protocol. While the contribution of the ciliary cleft to glaucoma pathogenesis has been more extensively investigated in dogs, comparable data in cats remain limited [19,20,21,22]. The normative values reported here may serve as a reference for future studies evaluating structural alterations in the ciliary cleft in feline secondary glaucoma and other anterior segment disorders.

The ciliary body parameters measured in this study, including Lf-CBT, LRf-CBT, CBAXL, and CPSA, provide insight into ciliary body morphology and potential functional dynamics. In prior feline UBM studies conducted by our group, CBAXL values ranging from 1.69 to 1.75 mm and CPSA values of approximately 77–79° were reported in clinically normal cats. In contrast, the present study demonstrated a larger mean CBAXL (2.01 mm) and a smaller CPSA (58.98°). An increase in CBAXL accompanied by a decrease in CPSA is consistent with a configuration suggestive of relative ciliary body contraction. These discrepancies are likely attributable to methodological differences among studies rather than true anatomical variation. Notably, UBM examinations in our previous studies were performed with cats positioned in sternal recumbency, whereas the present study was conducted with cats in dorsal recumbency. Differences in body positioning may influence ciliary body configuration through gravitational effects, probe orientation, and anterior segment geometry during image acquisition (Tables S1 and S2).

Although previous studies in dogs have demonstrated associations between ciliary muscle morphology and IOP, the clinical relevance of these parameters in cats remains uncertain, particularly given the relatively limited contribution of unconventional outflow to total aqueous humor drainage in this species. As such, these parameters should be interpreted primarily as descriptive reference values within a standardized anatomical framework [19,23].

Parameters describing the spatial relationships between the limbus, trabecular meshwork, iris, and ciliary processes—namely DLCP, TCPD, and ICPD—may be informative for assessing anterior segment configuration and aqueous outflow dynamics. In human studies, anterior positioning of the ciliary processes and reduced TCPD or ICPD have been associated with angle-closure mechanisms [1,7,24,25,26,27,28]. Although the pathophysiology of glaucoma differs between humans and cats, the reference values provided in this study may support future investigations into structural risk factors for feline secondary glaucoma.

Iris thickness parameters, including IBW and IMW, are particularly relevant in cats because differentiation between benign iris melanosis and feline diffuse iris melanoma can be challenging. Increases in IBW or IMW have been associated with tumor invasion and disease progression [29,30]. The normative values reported here are consistent with those of previous studies and may aid longitudinal monitoring and clinical decision-making in cats with iris pigmentation disorders.

The iris–lens angle (ILA) further characterizes the spatial relationship between the iris and lens and complements the assessment of overall iris configuration. However, ILA is known to be influenced by physiological changes in pupil size during mydriasis and miosis [31]. Because pharmacologic pupil dilation or constriction was not applied to standardize iris configuration in this study, variability in pupil dynamics among individual cats may have contributed to the relatively wide range observed for ILA measurements.

Although sex-related differences in anterior segment parameters have been associated with primary angle-closure glaucoma (PACG) risk in dogs, direct extrapolation of these findings to cats should be avoided [32,33,34]. In feline populations, the vast majority of glaucoma cases are secondary in origin, and when primary glaucoma occurs, it is most commonly classified as primary open-angle glaucoma (POAG) rather than PACG [35]. Primary angle-closure glaucoma is considered exceedingly rare in cats, with only a single case series reported in six Burmese cats [36]. Therefore, the sex-related differences observed in the present study should be interpreted as baseline anatomical variations rather than as indicators of PACG susceptibility in cats. These findings may nonetheless provide useful reference information for future investigations into feline anterior segment anatomy and glaucoma pathophysiology.

Body weight demonstrated significant positive correlations with iris thickness parameters, including IBW and IMW, whereas no meaningful associations were observed with most other anterior segment measurements. In general, larger body size might be expected to correspond with proportionally larger ocular dimensions. However, the limited influence of body weight on the majority of UBM parameters observed in the present study suggests that body weight alone may not accurately reflect overall body conformation in cats.

In the present study, no significant associations were identified between age and any UBM-derived anterior segment parameters. This finding suggests that anterior segment morphology remains relatively stable across the adult age range examined in cats. In contrast, previous studies in dogs have reported age-related anterior segment remodeling, including a gradual reduction in the ICA and an increased prevalence of pectinate ligament dysplasia, both of which may contribute to glaucoma development. However, comparable age-related changes were not observed in the feline eyes evaluated in the present study [37,38]. This discrepancy may reflect species-specific differences in anterior segment anatomy or glaucoma pathophysiology between dogs and cats, although further studies including older cats and diseased populations are required to confirm this observation.

Several limitations of this study should be acknowledged. First, only clinically normal cats were included, and eyes with ocular disease were not evaluated. Therefore, the clinical applicability of the reported reference values to pathological conditions should be interpreted cautiously. Second, only one eye per animal was included in the analysis to avoid intra-individual correlation and potential pseudoreplication. This approach was adopted to preserve statistical independence rather than to assume bilateral anatomical symmetry between eyes. Bilateral symmetry of UBM-derived anterior segment parameters was not formally assessed in this study and should be investigated in future studies using paired-eye analyses or intraclass correlation methods. In addition, formal assessments of measurement reproducibility, including inter-observer variability, intra-observer repeatability, intraclass correlation coefficients, or Bland–Altman analysis, were not performed. Although all measurements were obtained by a single trained examiner and mean values from multiple images were used to minimize measurement variability, formal reproducibility analyses would further strengthen the robustness and generalizability of the normative reference data. Finally, fundus examinations following pharmacologic pupil dilation were not performed prior to UBM imaging. Although neuro-ophthalmic examinations were normal in all cats and measurements were obtained under standardized non-mydriatic conditions, the possibility of undetected posterior segment abnormalities cannot be completely excluded.

## 5. Conclusions

In conclusion, this study established comprehensive normative UBM reference values for anterior segment structures in clinically normal cats under standardized imaging conditions. Most UBM-derived parameters were consistent across sex, body weight, and age, although limited associations were identified for specific iris-related and anterior chamber parameters. These findings provide a quantitative anatomical framework that may support future clinical and disease-oriented studies evaluating anterior segment alterations in feline ocular disorders.

## Figures and Tables

**Table 1:** Anterior Chamber Parameters Measured by Ultrasound Biomicroscopy.

Parameter	Description	Figure
Anterior chamber depth (ACD)	Distance between the corneal endothelium and the anterior surface of the lens.	Figure 1A
Peripheral anterior chamber depth (P-ACD)	Distance from the endpoint of Descemet’s membrane to the surface of the lens.	Figure 1B
Perilimbal corneal thickness (PCT)	Distance from the corneal epithelium at the conjunctival border (i.e., the limbal region) to the corneal endothelium	Figure 1B
Angle-opening distance (AOD)	Distance perpendicular from the endpoint of Descemet’s membrane to the iris.	Figure 1B
Iridocorneal angle (ICA)	The angle at the peripheral circumference of the anterior chamber where the sclera, cornea, and iris base converge.	Figure 1B
Ciliary cleft width (CCW)	Distance from where the outer layer of the pectinate ligament meets the inner boundary of the sclera to the upper surface of the iris root.	Figure 1C
Ciliary cleft length (CCL)	Distance from the angle recess to the midpoint of the ciliary cleft width.	Figure 1C
Ciliary cleft area (CCA)	Area formed by tracing the inner scleral surface from the angle recess to its inner boundary and the superior surface of the iris root, providing insights into the anatomical structure of the ciliary cleft.	Figure 1D
Longitudinal region of ciliary body thickness (Lf-CBT)	Measured by drawing a perpendicular line from the inner layer of the sclera, using the angle recess as a reference point, to where the line intersects the sclera and the iris root’s inner layers.	Figure 1E
Combined longitudinal and radial region of ciliary body thickness (LRf-CBT)	Measured by establishing a baseline along the inner layer of the sclera and another along the outer layer of the iris root, with a perpendicular line drawn from the inner layer of the sclera using the angle recess as a reference.	Figure 1E
Ciliary body axial length (CBAXL)	Distance between the apex of the dome-shaped ciliary body and the uveoscleral interface along the ciliary body’s long axis.	Figure 1F
Ciliary process scleral angle (CPSA)	Distance from the internal limbus to the first ciliary process.	Figure 1F
Distance from limbus to first ciliary process (DLCP)	Distance from the corneoscleral border to the point where a normal line is perpendicular to the iris and the ciliary process.	Figure 1G
Trabecular–ciliary process distance (TCPD)	Distance from the corneoscleral border to the point where a normal line is perpendicular to the iris and the ciliary process.	Figure 1G
Iris–ciliary process distance (ICPD)	Distance between the iris and the ciliary process along the line of TCPD	Figure 1G
Iris base width (IBW)	Thickness of the iris at the point where the pectinate ligaments meet the iris.	Figure 1H
Iris middle width (IMW)	Thickness of the iris at the midpoint between the posterior surface of the iris base and the point where the iris contacts the lens.	Figure 1H
Iris–lens angle (ILA)	Angle between the iris and the lens near the pupillary edge.	Figure 1H

**Table 2:** Mean and Range of Feline Anterior Segment Parameters Assessed by Ultrasound Biomicroscopy.

Parameter	Mean ± Std	Range	95% CI
Anterior chamber depth (ACD)	4.11 ± 0.32 mm	3.60–4.65 mm	3.96–4.26
Peripheral anterior chamber depth (P-ACD)	3.11 ± 0.21 mm	2.63–3.52 mm	3.01–3.21
Perilimbal corneal thickness (PCT)	590 ± 72 μm	350–680 μm	550–610
Angle-opening distance (AOD)	1.71 ± 0.40 mm	1.12–2.58 mm	1.52–1.89
Iridocorneal angle (ICA)	28.89 ± 4.40°	20.30–37.00°	26.8–30.92
Ciliary cleft width (CCW)	1.19 ± 0.16 mm	0.84–1.41 mm	1.12–1.27
Ciliary cleft length (CCL)	2.07 ± 0.23 mm	1.66–2.48 mm	1.96–2.18
Ciliary cleft area (CCA)	1.10 ± 0.25 mm^2^	0.65–1.54	0.98–1.22
Longitudinal region of ciliary body thickness (Lf-CBT)	0.48 ± 0.08 mm	0.36–0.62 mm	0.44–0.52
Longitudinal and radial region of ciliary body thickness (LRf-CBT)	0.76 ± 0.08 mm	0.61–0.88 mm	0.73–0.80
Ciliary body axial length (CBAXL)	2.04 ± 0.39 mm	1.19–2.56 mm	1.86–2.23
Ciliary process scleral angle (CPSA)	58.27 ± 11.97°	43.33–89.80°	52.67–63.87
Distance from limbus to first ciliary process (DLCP)	2.26 ± 0.58 mm	1.31–3.57 mm	1.99–2.53
Trabecular–ciliary process distance (TCPD)	2.35 ± 0.39 mm	1.58–2.82 mm	2.17–2.53
Iris–ciliary process distance (ICPD)	0.89 ± 0.24 mm	0.46–1.22 mm	0.78–1.00
Iris base width (IBW)	0.34 ± 0.06 mm	0.24–0.47 mm	0.32–0.37
Iris middle width (IMW)	0.53 ± 0.11 mm	0.35–0.73 mm	0.48–0.58
Iris–lens angle (ILA)	23.25 ± 7.44°	15.33–39.73°	19.77–26.73

## Data Availability

The original contributions presented in this study are included in the article/Supplementary Material. Further inquiries can be directed to the corresponding author(s).

## Supplementary Materials

The following supporting information can be downloaded at: https://www.mdpi.com/article/10.3390/vetsci13010050/s1, Table S1: Comparison of UBM methodologies and anterior segment measurements in normal cats; Table S2: Reference comparison of ultrasound biomicroscopy—derived anterior segment parameter values in clinically normal cats.

## Abbreviations

The following abbreviations are used in this manuscript:

UBM	Ultrasound biomicroscopy
PCT	Perilimbal corneal thickness
ACD	Anterior chamber depth
P-ACD	Peripheral anterior chamber depth
AOD	Angle-opening distance
ICA	Iridocorneal angle
CCW	Ciliary cleft width
CCL	Ciliary cleft length
CCA	Ciliary cleft area
Lf-CMT	Longitudinal fibers of ciliary muscle thickness
LRf-CMT	Longitudinal and radial fibers of ciliary muscle thickness
CBAXL	Ciliary body axial length
CPSA	Ciliary process scleral angle
DLCP	Distance from limbus to the first ciliary process
TCPD	Trabecular–ciliary process distance
ICPD	Iris–ciliary process distance
IBW	Iris base width
IMW	Iris middle width
ILA	Iris–lens angle
IOP	Intraocular pressure
FDIM	Feline diffuse iris melanoma
PACG	Primary angle-closure glaucoma
