# Supplementary material for: Anterior Segment Measurements in Normal Cats Using Ultrasound Biomicroscopy
Source: Vet Sci. 2026 Jan 6;13(1):50. doi: 10.3390/vetsci13010050 (PMC12846405; doi:10.3390/vetsci13010050)
Supplement: Supplementary file 1 [file vetsci-13-00050-s001.zip › Supplementary_Table_S2.docx]

**Supplementary Table S2. Reference comparison of ultrasound biomicroscopy–derived anterior segment parameter values in clinically normal cats**

Values are presented as mean ± SD as reported in each study. Only baseline measurements obtained from clinically normal cats prior to any pharmacologic intervention were included. Direct numerical comparisons across studies should be interpreted cautiously due to differences in study design, body position, and imaging protocols.

| **Parameter** | **Current study** | **PMID: 40102853** | **PMID: 41047838** | **PMID: 41047758** |
| --- | --- | --- | --- | --- |
| PCT (µm) | 590 ± 72 | - | - | - |
| ACD (mm) | 4.11 ± 0.32 | - | 4.52 ± 0.34 | - |
| P-ACD / SLD (mm) | 3.11 ± 0.21 | - | 3.09 ± 0.21 | - |
| AOD (mm) | 1.71 ± 0.40 | - | 1.83 ± 0.39 | 1.75 ± 0.36 |
| ICA (°) | 28.89 ± 4.40 | - | 30.27 ± 3.95 | 27.8 ± 4.1 |
| CCW (mm) | 1.19 ± 0.16 | - | 1.23 ± 0.24 | 1.18 ± 0.20 |
| CCL (mm) | 2.07 ± 0.23 | - | 2.04 ± 0.25 | 2.01 ± 0.22 |
| CCA (mm²) | 1.10 ± 0.25 | - | 1.15 ± 0.24 | 1.08 ± 0.21 |
| Lf-CBT (mm) | 0.48 ± 0.08 | - | - | - |
| LRf-CBT (mm) | 0.76 ± 0.08 | - | - | - |
| CBAXL (mm) | 2.04 ± 0.39 | - | - | 1.98 ± 0.31 |
| CPSA | 58.27 ± 11.97 | - | - | 76.5 ± 8.9 |
| DLCP (mm) | 2.26 ± 0.58 | - | - | - |
| TCPD (mm) | 2.35 ± 0.39 | - | - | - |
| ICPD (mm) | 0.89 ± 0.24 | - | - | - |
| IBW (mm) | 0.34 ± 0.06 | - | - | - |
| IMW (mm) | 0.53 ± 0.11 | - | - | - |
| ILA (°) | 23.25 ± 7.44 | - | - | - |

- = not reported. In PMID: 40102853, baseline UBM parameter values in normal cats were not explicitly reported as absolute numerical values; therefore, these fields are indicated as NR.
