# Supplementary material for: Anterior Segment Measurements in Normal Cats Using Ultrasound Biomicroscopy
Source: Vet Sci. 2026 Jan 6;13(1):50. doi: 10.3390/vetsci13010050 (PMC12846405; doi:10.3390/vetsci13010050)
Supplement: Supplementary file 1 [file vetsci-13-00050-s001.zip › Supplementary_Table_S1.docx]

**Supplementary Table S1. Comparison of UBM methodologies and anterior segment measurements in normal cats**

|  | **Current study** | **PMID: 40102853** | **PMID: 41047838** | **PMID: 41047758** |
| --- | --- | --- | --- | --- |
| **Study title** | Anterior Segment Measurements in Normal Cats Using Ultrasound Biomicroscopy | Effects of Omidenepag Isopropyl Versus Latanoprost Eye Drops on Intraocular Pressure, Pupil Diameter, and Anterior Chamber Parameters in Normal Feline Eyes: A Pilot Study | Comparative analysis of iridocorneal angle in cats and dogs using ultrasound biomicroscopy: implications for glaucoma prevalence | Ultrasound Biomicroscopic Study of the Effects of 1% Tropicamide on the Anterior Segment and Ciliary Body in Cats |
| **Study aim** | Normative reference | Drug-induced anterior chamber changes | Species comparison | Mydriasis-induced changes |
| **Study design** | Prospective | Prospective crossover | Retrospective | Prospective |
| **Animals (cats)** | 20 cats (20 eyes) | 22 cats (44 eyes) | 16 cats (16 eyes) | 14 cats (28 eyes) |
| **Sex (M/F)** | 9 / 11 | 12 / 10 | 8 / 8 | 7 / 7 |
| **Age (years)** | 5.5 ± 3.15 (2–13) | 4.95 (1–11) | 4.28 (1–9) | 4.79 ± 2.61 |
| **Body weight (kg)** | 5.32 ± 0.85 | Not specified | 5.18 (2.55–7.20) | 5.20 ± 0.82 |
| **Anesthesia** | General | General | General | General |
| **Body position** | Dorsal | Sternal | Sternal | Sternal |
| **Scan location** | 12-o’clock limbus | Superotemporal limbus | 12-o’clock limbus | 12-o’clock limbus |
